# Initial change in fractional excretion of total protein after SGLT2 inhibitors predicts renal prognosis in patients with chronic kidney disease

**DOI:** 10.1093/ckj/sfaf209

**Published:** 2025-07-07

**Authors:** Hideaki Kuno, Go Kanzaki, Rina Oba, Hirokazu Marumoto, Saeko Hatanaka, Takaya Sasaki, Kotaro Haruhara, Kei Matsumoto, Kentaro Koike, Hiroyuki Ueda, Yudo Tanno, Keita Hirano, Masato Ikeda, Nobuo Tsuboi, Takashi Yokoo

**Affiliations:** Division of Nephrology and Hypertension, Jikei University School of Medicine, Tokyo, Japan; Division of Nephrology and Hypertension, Jikei University School of Medicine, Tokyo, Japan; Division of Nephrology and Hypertension, Jikei University School of Medicine, Tokyo, Japan; Division of Nephrology and Hypertension, Jikei University School of Medicine, Tokyo, Japan; Division of Nephrology and Hypertension, Jikei University School of Medicine, Tokyo, Japan; Division of Nephrology and Hypertension, Jikei University School of Medicine, Tokyo, Japan; Division of Nephrology and Hypertension, Jikei University School of Medicine, Tokyo, Japan; Division of Nephrology and Hypertension, Jikei University School of Medicine, Tokyo, Japan; Division of Nephrology and Hypertension, Jikei University School of Medicine, Tokyo, Japan; Division of Nephrology and Hypertension, Jikei University School of Medicine, Tokyo, Japan; Division of Nephrology and Hypertension, Jikei University School of Medicine, Tokyo, Japan; Division of Nephrology and Hypertension, Jikei University School of Medicine, Tokyo, Japan; Division of Nephrology and Hypertension, Jikei University School of Medicine, Tokyo, Japan; Division of Nephrology and Hypertension, Jikei University School of Medicine, Tokyo, Japan; Division of Nephrology and Hypertension, Jikei University School of Medicine, Tokyo, Japan

**Keywords:** chronic kidney disease, fractional excretion of total protein, initial eGFR dip, renal prognosis, sodium–glucose co-transporter 2 inhibitors

## Abstract

**Background:**

Sodium–glucose co-transporter 2 inhibitors (SGLT2i) reduce glomerular hyperfiltration, resulting in an initial estimated glomerular filtration rate (eGFR) dip in chronic kidney disease (CKD). The association between the initial eGFR dip after SGLT2i and proteinuria reduction has not been explored. Fractional excretion of total protein (FETP) is an index of protein leakage corrected for GFR and may be useful in addressing this issue.

**Methods:**

FETP was calculated as (serum creatinine × urine protein)/(serum protein × urine creatinine) (in %) and the initial FETP dip was defined as 3 months/baseline FETP. The patients were divided into three groups according to the initial FETP dip tertile: FETP acute dipper, moderate dipper and riser. The association between initial FETP dip after SGLT2i and the eGFR slope over the subsequent 2 years was retrospectively investigated.

**Results:**

The study involved 238 patients, including 105 with diabetes mellitus. The patients’ median age was 57.0 years and eGFR was 43.0 ml/min/1.73 m^2^. The initial FETP dip was associated with the baseline eGFR, protein:creatinine ratio and FETP. The eGFR slope in the FETP acute dipper was −0.2 ml/min/1.73 m^2^/year, which was less than those of the moderate dipper and riser (−1.0 and −1.3 ml/min/1.73 m^2^/year; *P* < .001). Multivariate regression analyses revealed that the initial FETP dip was associated with the eGFR slope independent of potential confounders (*P* < .001).

**Conclusions:**

An FETP acute dip after SGLT2i is associated with a favourable renal prognosis in CKD, indicating that FETP is a useful index for assessing the efficacy of SGLT2i.

## Graphical Abstract

**Graphical Abstract:**
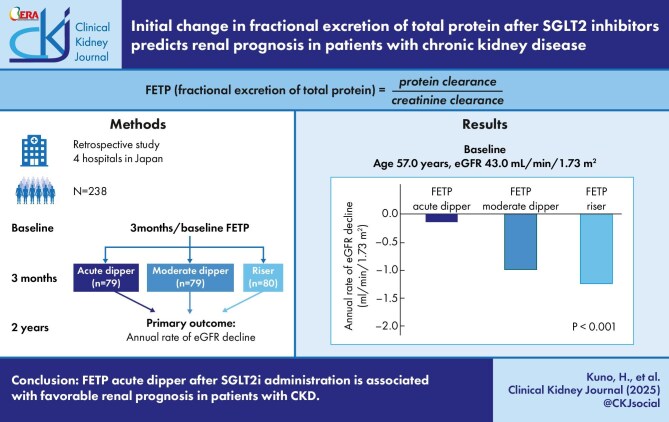

KEY LEARNING POINTS **What was known:**

- Sodium–glucose co-transporter 2 inhibitors (SGLT2i) reduce glomerular hyperfiltration, resulting in an initial eGFR dip in patients with CKD.
- The initial eGFR dip is associated with proteinuria reduction and renal prognosis.
- Fractional excretion of total protein (FETP) is an index of protein leakage corrected for GFR.

**This study adds:**

- The initial change in FETP following SGLT2i administration is associated with renal prognosis in CKD patients with and without diabetes mellitus.
- FETP can be used to assess the underlying hyperfiltration in patients with CKD.

**Potential impact:**

- FETP is a useful index of the efficacy of SGLT2i.
- FETP is a convenient marker that can be calculated using blood and urine tests and is useful for clinicians.

## INTRODUCTION

Sodium–glucose co-transporter 2 inhibitors (SGLT2i) reduce proteinuria and improve renal prognosis in patients with chronic kidney disease (CKD) [1–4]. SGLT2i reduces intraglomerular pressure through activation of tubuloglomerular feedback (TGF) and is known to be associated with an acute reduction in the estimated glomerular filtration rate (eGFR),

called the initial eGFR dip [5]. TGF is an adaptive mechanism that controls the single-nephron GFR in accordance with the salt concentration of the tubular fluid at the macula densa. SGLT2i suppresses salt and sugar reabsorption in the proximal tubules, which promotes sodium, chloride and water transport to the macula densa. The macula densa releases adenosine triphosphate (ATP) in proportion to the luminal solute concentration. ATP binds to purinergic P2 receptors on afferent arterioles or cleaves to release adenosine, resulting in afferent arteriolar vasoconstriction. Furthermore, the adenosine-mediated effects on juxtaglomerular cells reduces renin secretion and alleviates renin–angiotensin–aldosterone–mediated efferent arteriolar vasoconstriction. Increased afferent arterial tone and decreased efferent arterial tone lower intraglomerular pressure and are thought to be the mechanisms of the initial eGFR dip and proteinuria reduction associated with improved glomerular hyperfiltration [5–7].

It has been reported that SGLT2i dapagliflozin, empagliflozin and canagliflozin cause the initial eGFR dip [1–3]. Approximately 28–50% of patients with CKD exhibit an initial eGFR dip >10% of the baseline eGFR, and their subsequent trend in renal function is reportedly more gradual than that of patients with an initial eGFR dip of <10% or no initial eGFR dip [8–10]. Although the initial eGFR dip is an important response to SGLT2i therapy, its association with proteinuria reduction, a well-known indicator of renal prognosis, has not yet been investigated [11].

Fractional excretion of total protein (FETP), i.e. protein clearance divided by creatinine (Cr) clearance, is an index of protein leakage corrected for GFR [12, 13]. FETP is reportedly more useful than proteinuria alone as a predictor of renal prognosis in post-renal transplant recipients and patients with chronic glomerulopathy [12–18]. Therefore, FETP changes may more accurately represent improvement in intraglomerular pressure following SGLT2i administration. This study retrospectively investigated the association between the initial change in FETP and subsequent eGFR slope in patients with CKD treated with SGLT2i.

## MATERIALS AND METHODS

### Participants

This retrospective observational study included CKD outpatients who received SGLT2i therapy (dapagliflozin, empagliflozin or canagliflozin) from 2015 to 2022 at the Jikei University Hospital, Tokyo, Japan, and its affiliated hospitals (Jikei University Katsushika Medical Center, Jikei University Daisan Hospital, and Jikei University Kashiwa Hospital). The study protocol was approved by the Ethics Review Board of the Jikei University School of Medicine (36-348, 12464). Furthermore, the study followed the tenets of the Declaration of Helsinki. Because this was a retrospective cohort study, information on the research plan was proposed and an opportunity to opt out was provided, therefore individual informed consent was not required.

### Exclusion criteria

The study exclusion criteria were age <20 years, follow-up time <2 years, use of immunosuppressive drugs, switching from renin–angiotensin system (RAS) blockers or loop diuretics to SGLT2i, SGLT2i discontinuation, SGLT2i dose change, death or hospitalization during the observation period [19].

### Definition

CKD was defined as an eGFR <60 ml/min/1.73 m^2^ and/or a protein:creatinine ratio (PCR) >0.15 g/g Cr [20]. Diabetes mellitus (DM) was defined as having a haemoglobin A1c (HbA1c) of 6.5% (National Glycohemoglobin Standardization Program) or receiving medications or insulin therapy. The initial eGFR dip was calculated as the 3-month eGFR/baseline eGFR and the initial FETP dip was calculated as the 3-month FETP/baseline FETP [19, 21]. Follow-up eGFR and FETP were taken as the closest values to the 3-month time point within a window of 1–3 months after SGLT2i administration. The patients were divided into three groups according to the initial FETP dip tertile: FETP acute dipper, FETP moderate dipper and FETP riser.

### Outcome

The primary outcome was evaluated by the eGFR slope, which was defined as the annual rate of eGFR decline from 3 months to 2 years after SGLT2i therapy initiation [10, 22].

### Measurements

Demographic data, such as age, sex, systolic blood pressure (SBP), medical history (DM) and medication history [RAS blocker, calcium channel blocker (CCB), loop diuretics, mineralocorticoid receptor blocker (MRB), antihyperlipidaemic drugs, insulin and SGLT2i], were obtained from the patients’ medical records. Laboratory measurements included haemoglobin (Hb), total protein (TP), Cr, uric acid (UA), low-density lipoprotein cholesterol (LDL-C), eGFR and PCR. For Japanese patients, eGFR was calculated as eGFR (ml/min/1.73 m^2^) = 194 × Cr^−1.094^ × age^−0.287^ × (0.739 for women) [23], whereas FETP was calculated as (serum Cr × urine protein)/(serum protein × urine Cr) (in %) [12–14].

### Statistical analysis

Continuous variables were expressed as medians and interquartile ranges (IQRs) or numbers and percentages. Kruskal–Wallis and chi-squared tests were used to evaluate the differences in the continuous and categorical variables, respectively. Furthermore, the Jonckheere–Terpstra test was employed to detect trends in baseline characteristics and laboratory data according to the FETP tertile. We examined the risk factors for the annual rate of eGFR decline in a multiple regression analysis [22] adjusted for known risk factors such as sex; age; SBP; use of RAS blocker, CCB, loop diuretics, insulin or dapagliflozin 10 mg; DM; HbA1c; eGFR; PCR; Hb; UA; and initial eGFR dip [8–10]. Statistical significance was defined according to a two-sided *P*-value <.05. All statistical analyses were conducted using SPSS Statistics for Windows version 29.0 (IBM, Armonk, NY, USA).

## RESULTS

### Baseline characteristics of the patients

**Figure 1:**
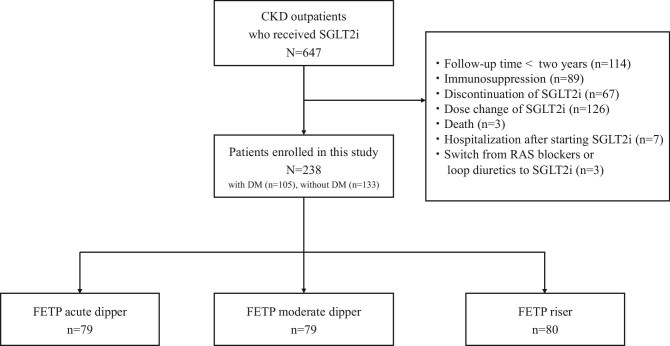
Patient flow and outcome. The patients were divided into three groups according to the initial FETP dip tertile.

During the study period, 647 outpatients with CKD were administered SGLT2i. Of them, 238 met the inclusion criteria (Fig. 1). The clinical and laboratory findings of the 238 patients are presented in Table 1. Their median age was 57.0 years (IQR 47.0–67.0) and 69.3% of them were men. Of the patients, 44.1% had DM and 88.2% used RAS blockers. The most frequently used SGLT2i was dapagliflozin (10 mg; 59.2%). Kidney biopsy was performed in 69.3% of patients and immunoglobulin A nephropathy (IgAN) accounted for 46.6% of the total biopsy cases. The median eGFR at diagnosis was 43.0 ml/min/1.73 m^2^ (IQR 31.3–55.3), TP 7.1 g/dl (IQR 6.7–7.5), PCR 0.7 g/g Cr (IQR 0.3–1.3) and FETP 0.010% (IQR 0.004–0.022).

**Table 1:** Baseline characteristics according to the initial FETP dip among the participants.

Factors	Overall (*N* = 238)	FETP acute dipper (*n* = 79)	FETP moderate dipper (*n* = 79)	FETP riser (*n* = 80)	*P*-value
Characteristics					
Age (years)	57.0 (47.0–67.0)	56.0 (48.0–64.0)	56.0 (47.0–67.0)	57.5 (47.0–69.0)	.434
Male, *n* (%)	165 (69.3)	54 (68.4)	51 (64.6)	60 (75.0)	.351
SBP (mmHg)^a^	124.0 (112.0–133.0)	120.0 (111.5–135.0)	124.5 (114.0–132.0)	125.0 (114.8–133.0)	.629
Diabetes mellitus, *n* (%)	105 (44.1)	42 (53.2)	33 (41.8)	30 (37.5)	.121
RAS blocker, *n* (%)	210 (88.2)	70 (88.6)	70 (88.6)	70 (87.5)	.969
MRB, *n* (%)	18 (7.6)	8 (10.1)	6 (7.6)	4 (5.0)	.474
CCB, *n* (%)	103 (43.3)	35 (44.3)	37 (46.8)	31 (38.8)	.574
Loop diuretic, *n* (%)	15 (6.3)	6 (7.6)	5 (6.3)	4 (5.0)	.797
Antihyperlipidaemic drug, *n* (%)	123 (51.7)	38 (48.1)	41 (51.9)	44 (55.0)	.684
Insulin therapy, *n* (%)	19 (8.0)	5 (6.3)	6 (7.6)	8 (10.0)	.686
SGLT2i, *n* (%)					
Dapagliflozin 5 mg	18 (7.6)	3 (3.8)	7 (8.9)	8 (10.0)	.013
Dapagliflozin 10 mg	141 (59.2)	39 (49.4)	48 (60.8)	54 (67.5)	
Empagliflozin 10 mg	42 (17.6)	17 (21.5)	17 (21.5)	8 (10.0)	
Canagliflozin 100 mg	37 (15.5)	20 (25.3)	7 (8.9)	10 (12.5)	
Cause of kidney disease, *n* (%)					
IgAN	111 (46.6)	30 (38.0)	40 (50.6)	41 (51.3)	.830
FSGS	19 (8.0)	7 (8.9)	9 (11.4)	3 (3.8)	
Benign nephrosclerosis	10 (4.2)	3 (3.8)	3 (3.8)	4 (5.0)	
Membranous nephropathy	7 (2.9)	2 (2.5)	3 (3.8)	2 (2.5)	
IgA vasculitis	5 (2.1)	2(2.5)	2 (2.5)	1 (1.3)	
ORG	3 (1.3)	1 (1.3)	1 (1.3)	1 (1.3)	
Other^b^	10 (4.2)	4 (5.1)	3 (3.8)	3 (3.8)	
Unknown	73 (30.7)	30 (38.0)	18 (22.8)	25 (31.3)	
Laboratory data					
Hb (g/dl)^a^	13.9 (12.7–15.3)	14.3 (12.8–15.6)	13.3 (12.0–14.8)	14.0 (13.1–15.2)	.864
TP (g/dl)	7.1 (6.7–7.5)	7.1 (6.7–7.5)	7.1 (6.7–7.5)	7.1 (6.7–7.4)	.856
Cr (mg/dl)	1.3 (1.0–1.8)	1.2 (0.9–1.6)	1.4 (1.1–2.0)	1.4 (1.1–1.8)	.024
eGFR (ml/min/1.73 m^2^)	43.0 (31.3–55.3)	46.0 (36.2–65.0)	38.0 (25.0–53.0)	42.6 (30.4–51.0)	.046
UA (mg/dl)^a^	6.4 (5.6–7.3)	6.5 (5.8–7.3)	6.5 (5.6–7.4)	6.3 (5.5–7.0)	.239
LDL-C (mg/dl)^a^	110.0 (92.0–133.0)	114.5 (95.0–135.8)	109.5 (90.3–134.5)	108.0 (90.0–131.0)	.280
HbA1c (%)^a^	6.1 (5.6–7.4)	6.6 (5.7–7.7)	6.1 (5.6–7.3)	6.0 (5.5–6.9)	.008
PCR (g/g Cr)	0.7 (0.3–1.3)	0.9 (0.4–1.7)	0.7 (0.4–1.4)	0.4 (0.2–1.1)	.002
FETP (%)	0.010 (0.004–0.022)	0.014 (0.006–0.028)	0.014 (0.008–0.033)	0.009 (0.003–0.022)	.041
Initial eGFR dip	0.94 (0.89–1.00)	0.90 (0.86–0.95)	0.94 (0.90–1.00)	0.98 (0.91–1.04)	<.001

Values are presented as median (IQR) unless stated otherwise.

FSGS: focal segmental glomerulosclerosis; ORG: obesity-related glomerulopathy.

a Number of missing values: body mass index, *n* = 22; SBP, *n* = 3; Hb, *n* = 1; UA, *n* = 12; LDL-C, *n* = 14.

b Other: Alport syndrome, diabetic nephropathy, Fabry disease, minimal change nephrotic syndrome, membranoproliferative glomerulonephritis, IgG4-related disease, chronic glomerular nephritis, malignant nephrosclerosis and eosinophilic granulomatosis with polyangiitis.

### Baseline characteristics and primary outcome by initial FETP dip category

**Figure 2:**
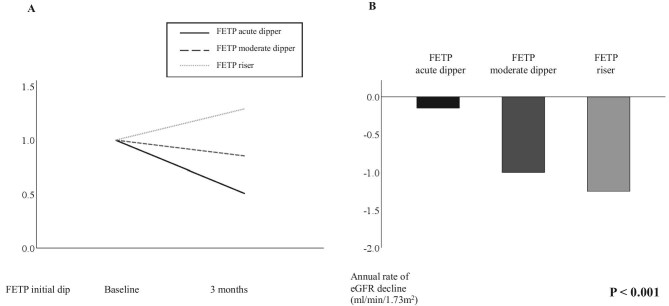
**(A)** Relative initial change in FETP when the baseline FETP is set to 1.0 and **(B)** annual rate of eGFR decline according to the initial FETP dip among all patients (*n* = 238).

**Figure 3:**
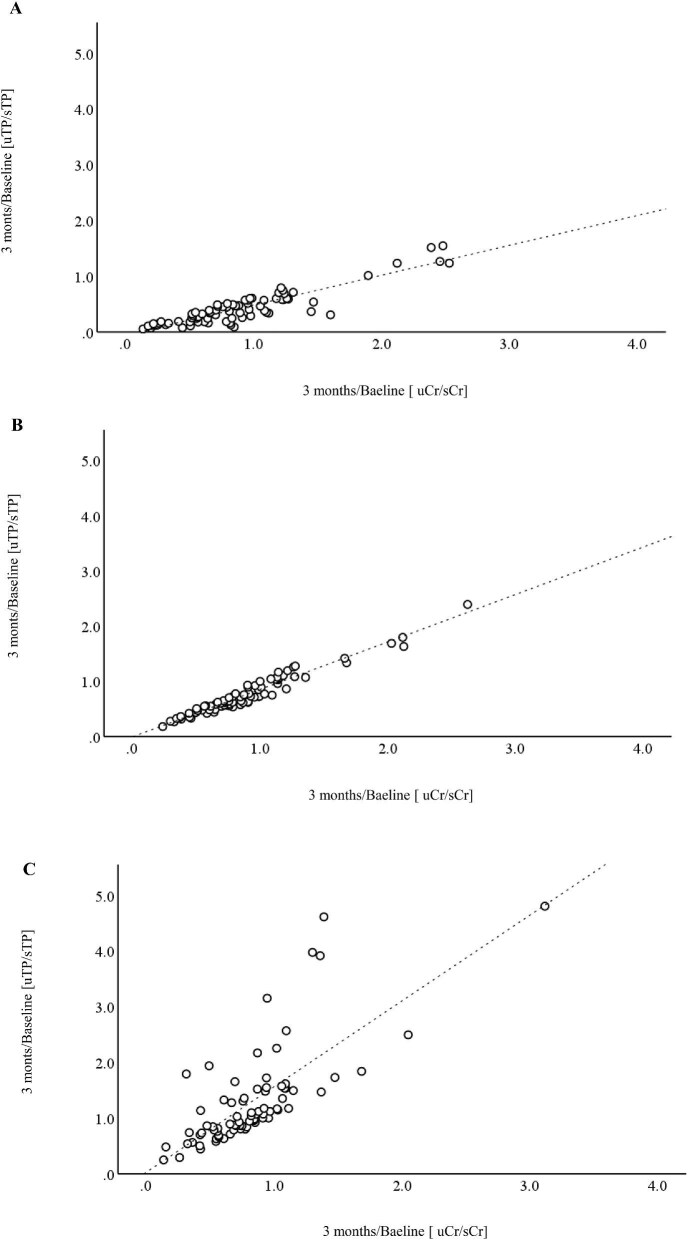
Scatter plot between 3 months/baseline (uCr/sCr) and 3 months/baseline (uTP/sTP) in **(A)** FETP acute dippers, **(B)** FETP moderate dippers and **(C)** FETP risers. (A) 3 months/baseline (uTP/sTP) = 0.54*3 months/baseline (uCr/sCr) − 0.05, *r* = 0.90 (95% CI 0.48–0.59), *P* < .001. (B) 3 months/baseline (uTP/sTP) = 0.86*3 months/baseline (uCr/sCr) − 6.21, *r* = 0.97 (95% CI 0.81–0.91), *P* < .001. (C) 3 months/baseline (uTP/sTP) = 1.54*3 months/baseline (uCr/sCr) + 0.03, *r* = 0.74 (95% CI 1.22–1.86), *P* < .001. uCR: urine creatinine; sCR: serum creatinine; uTP: urine total protein; sTP: serum total protein.

The HbA1c values were significantly higher in the FETP acute dipper group. Empagliflozin and canagliflozin were used more frequently in this group. The baseline eGFR, PCR and FETP were higher in the FETP acute dipper group than in the FETP moderate dipper and FETP riser groups. Better renal prognosis was observed in the FETP acute dipper group, with an eGFR slope of −0.2 ml/min/1.73 m^2^/year (IQR −1.5–2.5) compared with −1.0 ml/min/1.73 m^2^/year (IQR −2.0–1.0) in the FETP moderate dipper group and −1.3 ml/min/1.73 m^2^/year (IQR −3.1 to −0.2) in the FETP riser group (*P* < .001) (Fig. 2 and Supplementary Table 1). Fig. 3 presents scatter plots between 3 months/baseline (urine Cr/serum Cr) and 3 months/baseline (urine TP/serum TP) to compare the behaviour of Cr clearance and protein clearance among the three groups. FETP acute dippers showed a greater reduction in protein clearance than Cr clearance.

### Baseline characteristics and primary outcomes of patients with or without DM by initial FETP dip category

We also investigated the baseline characteristics and primary outcomes of patients with DM (*n* = 105) and those without DM (*n* = 133) (Table 2). Empagliflozin and canagliflozin were more frequently used by patients with DM, whereas dapagliflozin was more frequently used by those without DM. In both patient groups, no significant association was observed between the type of SGLT2i and initial FETP dip.

**Table 2:** Baseline characteristics according to the initial FETP dip in patients with and without DM.

Factors	Patients with DM (*N* = 105)	FETP acute dipper (*n* = 35)	FETP moderate dipper (*n* = 35)	FETP riser (*n* = 35)	*P*-value	Patients without DM (*N* = 133)	FETP acute dipper (*n* = 44)	FETP moderate dipper (*n* = 44)	FETP riser (*n* = 45)	*P*-value
Characteristics										
Age (years)	59.0 (50.0–68.5)	58.0 (48.0–64.0)	59.0 (51.0–67.0)	66.0 (54.0–74.0)	.009	54.0 (45.0–65.5)	54.5 (43.5–65.5)	55.5 (47.0–68.8)	52.0 (43.0–62.0)	.464
Male, *n* (%)	79 (75.2)	24 (68.6)	28 (80.0)	27 (77.1)	.515	86 (64.7)	26 (59.1)	28 (63.6)	32 (71.1)	.487
SBP (mmHg)^a^	125.0 (115.0–135.0)	127.0 (114.3–135.8)	128.0 (114.0–146.0)	123.5 (115.8–133.0)	.621	123.0 (110.0–132.0)	118.5 (109.5–130.5)	124.0 (110.0–135.0)	124.0 (110.0–132.0)	.385
RAS blocker, *n* (%)	91 (86.7)	30 (85.7)	32 (91.4)	29 (82.9)	.562	119 (89.5)	41 (93.2)	38 (86.4)	40 (88.9)	.574
MRB, *n* (%)	6 (5.7)	4 (11.4)	2 (5.7)	0 (0.0)	.120	12 (9.0)	3 (6.8)	5 (11.4)	4 (8.0)	.758
CCB, *n* (%)	59 (56.2)	21 (60.0)	22 (62.9)	16 (45.7)	.301	44 (33.1)	13 (29.5)	16 (36.4)	15 (33.3)	.793
Loop diuretic, *n* (%)	8 (7.6)	2 (5.7)	4 (11.4)	2 (5.7)	.582	7 (5.3)	2 (4.5)	3 (6.8)	2 (4.4)	.852
Antihyperlipidaemic drug, *n* (%)	64 (61.0)	22 (62.9)	21 (60.0)	21 (60.0)	.961	59 (44.4)	17 (38.6)	21 (47.7)	21 (46.7)	.643
Insulin therapy, *n* (%)	19 (18.1)	4 (11.4)	7 (20.0)	8 (22.9)	.434	0 (0.0)	0 (0.0)	0 (0.0)	0 (0.0)	–
SGLT2i, *n* (%)										
Dapagliflozin 5 mg	11 (10.5)	2 (5.7)	3 (8.6)	6 (17.1)	.226	7 (5.3)	1 (2.3)	3 (6.8)	3 (6.7)	.322
Dapagliflozin 10 mg	19 (18.1)	5 (14.3)	6 (17.1)	8 (22.9)		122 (91.7)	40 (90.9)	41 (93.2)	41 (91.1)	
Empagliflozin 10 mg	38 (36.2)	11 (31.4)	17 (48.6)	10 (28.6)		4 (3.0)	3 (6.8)	0 (0.0)	1 (2.2)	
Canagliflozin 100 mg	37 (35.2)	17 (48.6)	9 (25.7)	11 (31.4)		0 (0.0)	0 (0.0)	0 (0.0)	0 (0.0)	
Cause of kidney disease, *n* (%)										
IgAN	22 (21.0)	7 (20.0)	7 (20.0)	8 (22.9)	.876	89 (66.9)	27 (61.4)	32 (72.7)	30 (66.7)	.774
FSGS	10 (9.5)	2 (5.7)	6 (17.1)	2 (5.7)		9 (6.8)	5 (11.4)	3 (6.8)	1 (2.2)	
Benign nephrosclerosis	7 (6.7)	2 (5.7)	3 (8.6)	2 (5.7)		3 (2.3)	1 (2.3)	0 (0.0)	2 (4.4)	
Membranous nephropathy	4 (3.8)	1 (2.9)	1 (2.9)	2 (5.7)		3 (2.3)	1 (2.3)	1 (2.3)	1 (2.2)	
IgA vasculitis	2 (1.9)	0 (0.0)	1 (2.9)	1 (2.9)		3 (2.3)	2 (4.5)	1 (2.3)	0 (0.0)	
ORG	1 (1.0)	1 (1.0)	0 (0.0)	0 (0.0)		2 (1.5)	0 (0.0)	1 (2.3)	1 (2.2)	
Other^b^	5 (4.8)	2 (5.7)	2 (5.7)	1 (2.9)		5 (3.8)	1 (2.3)	2 (4.5)	2 (4.4)	
Unknown	54 (51.4)	20 (57.1)	15 (42.9)	19 (54.3)		19 (14.3)	7 (15.9)	4 (9.1)	8 (17.8)	
Laboratory data										
Hb (g/dl)^a^	14.4 (12.9–15.7)	14.8 (13.4–15.7)	14.1 (12.4–16.0)	14.0 (12.9–15.4)	.398	13.6 (12.3–15.0)	13.2 (12.3–14.7)	13.2 (12.0–15.2)	14.1 (13.3–15.2)	.100
TP (g/dl)	7.2 (6.9–7.6)	7.2 (6.9–7.5)	7.3 (6.7–7.6)	7.2 (6.9–7.6)	.618	7.0 (6.6–7.2)	6.9 (6.5–7.2)	7.1 (6.8–7.5)	6.8 (6.6–7.2)	.909
Cr (mg/dl)	1.2 (0.9–1.6)	1.1 (0.9–1.3)	1.2 (1.0–2.0)	1.2 (1.0–1.8)	.043	1.4 (1.1–1.8)	1.4 (1.0–1.7)	1.5 (1.2–2.1)	1.4 (1.1–1.8)	.707
eGFR (ml/min/1.73 m^2^)	49.0 (32.3–62.0)	56.0 (41.0–69.2)	50.0 (24.0–56.0)	45.0 (32.0–51.0)	.026	40.0 (29.0–48.5)	40.5 (32.3–47.8)	35.5 (21.3–47.3)	42.2 (30.9–52.0)	.820
UA (mg/dl)^a^	6.3 (5.6–7.2)	6.5 (5.8–7.4)	6.4 (5.6–7.2)	6.2 (5.2–7.1)	.257	6.5 (5.7–7.3)	6.6 (5.7–7.3)	6.8 (5.7–7.5)	6.2 (5.5–7.0)	.162
LDL-C (mg/dl)^a^	114.0 (95.1–131.5)	125.5 (104.3–147.3)	107.5 (86.8–126.0)	108.4 (94.0–130.2)	.065	108.0 (90.0–137.0)	105.0 (90.0–132.0)	118.8 (95.8–144.5)	107.0 (84.0–136.8)	.914
HbA1c (%)	7.4 (6.8–8.1)	7.5 (6.9–8.1)	7.5 (7.1–8.2)	6.9 (6.7–7.8)	.143	5.6 (5.4–5.9)	5.6 (5.4–6.0)	5.6 (5.4–5.9)	5.6 (5.4–5.9)	.589
PCR (g/g Cr)	0.7 (0.3–1.7)	0.9 (0.3–1.7)	1.1 (0.6–2.4)	0.3 (0.1–1.3)	.062	0.6 (0.4–1.2)	0.8 (0.5–1.3)	0.7 (0.4–1.2)	0.4 (0.2–1.1)	.051
FETP (%)	0.011 (0.004–0.026)	0.016 (0.006–0.032)	0.007 (0.001–0.022)	0.007 (0.001–0.022)	.149	0.012 (0.006–0.030)	0.007 (0.013–0.029)	0.014 (0.007–0.030)	0.010 (0.004–0.024)	.173
Initial eGFR dip	0.94 (0.88–1.00)	0.92 (0.85–0.99)	0.94 (0.87–0.98)	0.98 (0.90–1.06)	.006	0.94 (0.89–1.00)	0.90 (0.88–0.96)	0.94 (0.89–1.00)	0.98 (0.91–1.03)	<.001

Values are presented as median (IQR) unless stated otherwise.

FSGS: focal segmental glomerulosclerosis; ORG: obesity-related glomerulopathy.

a Number of missing values: *n* = 12; SBP, *n* = 1; Hb, *n* = 1; UA, *n* = 8; LDL-C, *n* = 1.

b Other: Alport syndrome, diabetic nephropathy, Fabry disease, minimal change nephrotic syndrome, membranoproliferative glomerulonephritis, IgG4-related disease, chronic glomerular nephritis, malignant nephrosclerosis and eosinophilic granulomatosis with polyangiitis.

Among patients with DM, the baseline eGFR was significantly higher in the FETP acute dipper group. Furthermore, the baseline PCR and FETP levels were higher in the FETP acute dipper group, although the difference was not significant. The annual eGFR decline was more gradual in the FETP acute dipper group [0.0 ml/min/1.73 m^2^/year (IQR −2.5–3.0)] than in the FETP moderate dipper and FETP riser groups [−1.0 ml/min/1.73 m^2^/year (IQR −2.0–2.0) and −1.5 ml/min/1.73 m^2^/year (IQR −2.5–0.0), respectively] (*P* = .042) (Supplementary Fig. 1 and Supplementary Table 2).

In patients without DM, the baseline eGFR, PCR and FETP were not significantly associated with the initial FETP dip. The FETP acute dipper group had a favourable renal prognosis [−0.5 ml/min/1.73 m^2^/year (IQR −1.5–1.3)] than the FETP moderate dipper and FETP riser groups [−0.5 ml/min/1.73 m^2^/year (IQR −1.5–1.0)] and −1.5 ml/min/1.73 m^2^/year (IQR −3.5 to −0.5), respectively] (*P* = .003) (Supplementary Fig. 2 and Supplementary Table 3).

### Multivariable regression analyses of factors associated with the rate of eGFR decline

Multivariable regression analyses were conducted to determine whether initial FETP dip was associated with the annual rate of eGFR decline (Table 3). We conducted a multivariate analysis adjusting for known risk factors for renal prognosis, such as sex; age; SBP, use of RAS blocker, CCB, loop diuretics and dapagliflozin 10 mg; DM; HbA1c use; eGFR; PCR; Hb; UA and initial eGFR dip (basic model), and showed that the initial FETP dip was an independent predictor of the annual rate of eGFR decline (*P* < .001).

Furthermore, stratified analysis by DM group, non-DM group and patients with IgAN revealed that the association between the initial FETP dip and renal prognosis remained unchanged.

Finally, we examined the effect of the initial FETP dip on the accuracy of the risk assessment of eGFR decline. We evaluated the difference in model fit when the initial FETP dip was added to the basic model consisting of the aforementioned factors that included the eGFR initial dip (Table 4). The addition of the information on the initial FETP dip to the basic model increased the *R*^2^ (from 0.26 to 0.31, *F* change = 13.4, *P* < .001).

**Table 3:** Predictors of annual eGFR decline.

	Univariable			Age- and sex-adjusted model			Multivariable adjusted model^a^		
Variables	95% CI	β	*P*-value	95% CI	β	*P*-value	95% CI	β	*P*-value
Initial FETP dip in overall patients (*n* = 238)	−0.044 to −0.018	−0.298	<.001	−0.044 to −0.018	−0.296	<.001	−0.040 to −0.016	−0.285	<.001
Initial FETP dip in patients with DM (*n* = 105)	−0.059 to −0.011	−0.278	.004	−0.066 to −0.019	−0.332	<.001	−0.057 to −0.012	−0.299	.003
Initial FETP dip in patients without DM (*n* = 133)	−0.043 to −0.014	−0.326	<.001	−0.044 to −0.015	−0.337	<.001	−0.041 to −0.011	−0.306	<.001
Initial FETP dip in patients with IgAN (*n* = 111)	−0.045 to −0.013	−0.322	<.001	−0.045 to −0.013	−0.323	<.001	−0.048 to −0.014	−0.351	<.001

CI: confidence interval.

a Adjusted for male sex, age, SBP, RAS blocker, CCB, loop diuretic, dapagliflozin 10 mg (versus dapagliflozin 5 mg/empagliflozin/canagliflozin), DM, HbA1c, eGFR, PCR, Hb, UA and initial eGFR dip.

**Table 4:** Predictive ability of the initial FETP dip in overall patients.

Model	*R*	*R* ^2^	Standard error	*F* change	*P*-value
Basic model	0.49	0.26	0.058	13.4	<.001
Basic model + initial FETP dip	0.53	0.31	0.056		

Basic model: adjusted for sex, age, SBP, RAS blocker, CCB, loop diuretic, antihyperlipidaemic drug, dapagliflozin 10 mg (versus dapagliflozin 5 mg/empagliflozin/canagliflozin), DM, HbA1c, eGFR, PCR, Hb, UA and initial eGFR dip.

## DISCUSSION

This study showed that in patients with CKD, a great reduction in FETP after SGLT2i administration was associated with a favourable renal prognosis, as indicated by the eGFR slope. Multivariate analysis revealed that the initial FETP dip was a predictor of the eGFR slope for 2 years independent of potential confounders. Furthermore, the study demonstrated that the initial FETP dip independently predicted the eGFR slope in stratified analyses based on the presence or absence of DM. To the best of our knowledge, this study is the first to explore the usefulness of initial FETP dip evaluation after SGLT2i administration in predicting the long-term renal prognosis of patients with CKD.

The concept of indexing proteinuria to renal function has also been supported by previous studies. Indexing proteinuria to eGFR may allow for a more accurate assessment of filtration barrier disease [24]. FETP is an indicator of protein clearance relative to Cr clearance and can be determined through routine blood and urine tests. The difference between FETP and index proteinuria against eGFR is the inclusion of serum TP in the equation. Therefore, FETP may be more sensitive to protein clearance changes. A previous study reported that the median FETP of 219 renal transplant recipients 1 year after transplantation was 0.0035%. In that study, a high FETP was an independent predictor of transplant failure and mortality and was shown to be more useful than PCR [12]. In nephrotic syndrome, FETP has been reported to better correlate with 24-h proteinuria than PCR [13]. Recently we reported a median FETP of 0.08% at the time of kidney biopsy in patients with primary membranous nephropathy (median eGFR 65.7 ml/min/1.73 m^2^; median PCR 5.3 g/g Cr). Elevated FETP was associated with severe proteinuria, advanced renal dysfunction and progressive kidney biopsy findings on electron microscopy, such as advanced global glomerulosclerosis and Ehrenreich–Churg stage, and with poor long-term renal prognosis [14]. These findings indicate that FETP is strongly associated with the severity and prognosis of renal diseases. However, the utility of FETP in the therapeutic monitoring of drugs has not yet been investigated.

SGLT2i can reduce intraglomerular pressure and suppress glomerular hyperfiltration by vasoconstricting the glomerular afferent arterioles through TGF [6, 7]. The suppression of glomerular hyperfiltration led to an initial eGFR dip after SGLT2i administration. Consistent with this idea, we have recently reported a greater initial eGFR dip in CKD patients treated with dapagliflozin who exhibited glomerular hypertrophy, a morphometric surrogate of glomerular hyperfiltration in kidney biopsy [25].

Previous studies that investigated the renal protective effect of SGLT2i have evaluated the initial eGFR dip and proteinuria, although the interactive kinetics of GFR and proteinuria have not been reported. This study focused on the initial change in FETP following SGLT2i administration to determine the association of proteinuria with GFR. The FETP acute dipper group had a greater reduction in protein clearance relative to Cr clearance with SGLT2i. Furthermore, the baseline FETP in the FETP acute dipper group was higher than that in the FETP riser group, indicating that the proteinuria in the former group was mediated by glomerular hyperfiltration. The FETP acute dipper group had urinary protein derived from hyperfiltration and therefore gained greater benefit from SGLT2i. In contrast, the patients in the FETP riser group may have had fragile kidneys in which the compensatory mechanism of hyperfiltration had failed. This group may have also included patients in whom osmotic diuresis caused an initial eGFR dip but had little effect on proteinuria inhibition. By observing changes in FETP, we can determine whether patients have hyperfiltration and whether SGLT2i exerts a significant renoprotective effect.

The initial FETP dip was associated with SGLT2i type and high HbA1c level in the entire study cohort. Therefore, we divided the cohort into DM and non-DM groups. An association between the initial FETP dip and high eGFR was observed in the DM group but not in the non-DM group. In patients with diabetic nephropathy, an absolute increase in GFR is often observed in the early stage of the disease, representing glomerular hyperfiltration at the whole-kidney level [26]. Therefore, the initial FETP dip may be useful in assessing suppression of whole-kidney hyperfiltration in patients with DM. Meanwhile, the present study did not observe any association between eGFR and initial FETP dip in the non-DM group. When nephrons are lost in patients with advanced CKD, the remaining glomeruli are compensated for by glomerular hyperfiltration at the single-nephron level to maintain normal levels of the total filtration function of the kidney [26]. Taken together with the previously reported findings, the results of the present study indicate that the initial FETP dip may differentially reflect changes in hyperfiltration at the whole-kidney or single-nephron level in patients with and without DM.

Multiple regression analysis showed that the model that considered the initial FETP dip in addition to the initial eGFR dip had a better fit and was an independent predictor of eGFR decline. These findings indicate that for similar degrees of initial eGFR dip, patients with better proteinuria suppression have better renal outcomes following SGLT2i treatment.

The DAPA-CKD study (NCT03036150) reported the effect of SGLT2i in IgAN on the improvement of proteinuria and renal outcomes [27]. Approximately half of the patients in the present study had IgAN. Thus we examined the association between the initial FETP dip and the eGFR slope in the subgroup analysis of patients with IgAN. We excluded cases of immunosuppressant use and major drug changes, which enabled us to accurately evaluate the effects of SGLT2i. Multiple regression analysis of this subgroup revealed that the initial FETP dip was an independent predictor of the eGFR slope, suggesting that the initial FETP dip is applicable to patients with IgAN in predicting long-term renal prognosis.

This study had several limitations. First, due to the retrospective nature of the study, we could not assess the body mass index before and after the initiation of SGLT2i therapy, which had a large number of missing values. Second, the observation period was only 2 years. Therefore, we were unable to evaluate renal prognosis for longer periods of time. However, over the 2-year observation period, the patients were more frequently treated with additional or changed medications. This represents bias when assessing the impact of SGLT2i on renal prognosis. Moreover, the median observation period in the previously reported randomized controlled trial was ≈2 years. Therefore, the observation period was sufficient to evaluate the effects of SGLT2i. Finally, all patients included in this study were Japanese. Thus the results cannot be generalized to patients of other races or from other regions.

In conclusion, the present study showed that the initial FETP dip after SGLT2i initiation was associated with the subsequent eGFR slope in CKD patients with and without DM. FETP may be a convenient and useful indicator for the response to therapy and subsequent long-term renal prognosis in patients with CKD treated with SGLT2i.

## Supplementary Material

sfaf209_Supplemental_Files

## Data Availability

The datasets used and/or analysed during the current study are available from the corresponding author upon reasonable request.
