# Supplementary material for: Initial change in fractional excretion of total protein after SGLT2 inhibitors predicts renal prognosis in patients with chronic kidney disease
Source: Clin Kidney J. 2025 Jul 7;18(8):sfaf209. doi: 10.1093/ckj/sfaf209 (PMC12319533; doi:10.1093/ckj/sfaf209)
Supplement: sfaf209_Supplemental_Files [file sfaf209_supplemental_files.zip › Supplementary Table1-4.pdf]

Supplementary Table 1. Initial FETP dip and annual rate of eGFR decline among all participants

	Overall<br>*N* = 238	FETP<br>acute dipper<br>n =79	FETP<br>moderate dipper<br>n = 79	FETP<br>riser<br>n = 80	P
Initial FETP dip	0.9<br>(0.6-1.2)	0.5<br>(0.4-0.6)	0.9<br>(0.8-0.9)	1.3<br>(1.2-1.7)	< 0.001
Annual rate of eGFR decline<br>(mL/min/1.73m^2/year)	-0.7<br>(-2.0 to 1.0)	-0.2<br>(-1.5 to 2.5)	-1.0<br>(-2.0 to 1.0)	-1.3<br>(-3.1 to -0.2)	< 0.001

Supplementary Table 2. Initial FETP dip and annual rate of eGFR decline among patients with DM

	Patients<br>with DM<br>*N* = 105	FETP<br>acute dipper<br>n =35	FETP<br>moderate dipper<br>n = 35	FETP<br>riser<br>n = 35	P
Initial FETP dip	0.8<br>(0.5-1.1)	0.5<br>(0.4-0.6)	0.8<br>(0.7-0.9)	1.2<br>(1.1-1.6)	< 0.001
Annual rate of eGFR decline<br>(mL/min/1.73m^2/year)	-1.0<br>(-2.5 to 2.0)	0.0<br>(-2.5 to 3.0)	-1.0<br>(-2.0 to 2.0)	-1.5<br>(-2.5 to 0.0)	0.042

Supplementary Table 3. Initial FETP dip and annual rate of eGFR decline among patients without DM

	Patient<br>without DM<br>*N* = 133	FETP<br>acute dipper<br>n =44	FETP<br>moderate dipper<br>n = 44	FETP<br>riser<br>n = 45	P
Initial FETP dip	0.9<br>(0.6-1.2)	0.5<br>(0.4-0.6)	0.9<br>(0.8-0.9)	1.4<br>(1.2-1.8)	< 0.001
Annual rate of eGFR decline<br>(mL/min/1.73m^2/year)	-0.5<br>(-2.0 to 0.5)	-0.5<br>(-1.5 to 1.3)	-0.5<br>(-1.5 to 1.0)	-1.5<br>(-3.5 to -0.5)	0.003

Supplementary Table 4. Predictors of annual eGFR decline

	Univariable		
	95% CI	P value	β
Male	-0.023 to 0.012	0.529	-0.041
Age	-0.015 to 0.048	0.306	0.067
Systolic blood pressure	-0.001 to 0.000	0.106	-0.110
RAS blockers	-0.013 to 0.036	0.356	0.060
Calcium channel blocker	-0.026 to 0.006	0.232	-0.078
Loop diuretics	-0.017 to 0.048	0.348	0.061
Dapagliflozin 10mg	-0.020 to 0.012	0.611	-0.033
(vs Dapagliflozin 5mg / Empagliflozin / Canagliflozin)			
DM	-0.011 to 0.021	0.556	0.038
HbA1c	-0.035 to 0.056	0.642	0.031
eGFR	-0.009 to 0.025	0.332	0.063
Hb	-0.008 to 0.104	0.109	0.096
UA	-0.049 to 0.026	0.541	-0.040
PCR	-0.013 to 0.000	0.057	-0.123
initial eGFR dip	-0.316 to -0.169	<0.001	-0.391
initial FETP dip	-0.044 to -0.018	<0.001	-0.298

Abbreviations: RAS blockers, renin-angiotensin system blockers; DM, diabetes mellitus; eGFR, estimated glomerular filtration rate; PCR, protein creatinine ratio; Hb, hemoglobin; UA, uric acid; FETP, fractional excretion of total protein
