# Supplementary figures and images for: Initial change in fractional excretion of total protein after SGLT2 inhibitors predicts renal prognosis in patients with chronic kidney disease
Source: Clin Kidney J. 2025 Jul 7;18(8):sfaf209. doi: 10.1093/ckj/sfaf209 (PMC12319533; doi:10.1093/ckj/sfaf209)

Supplementary Figure 1a

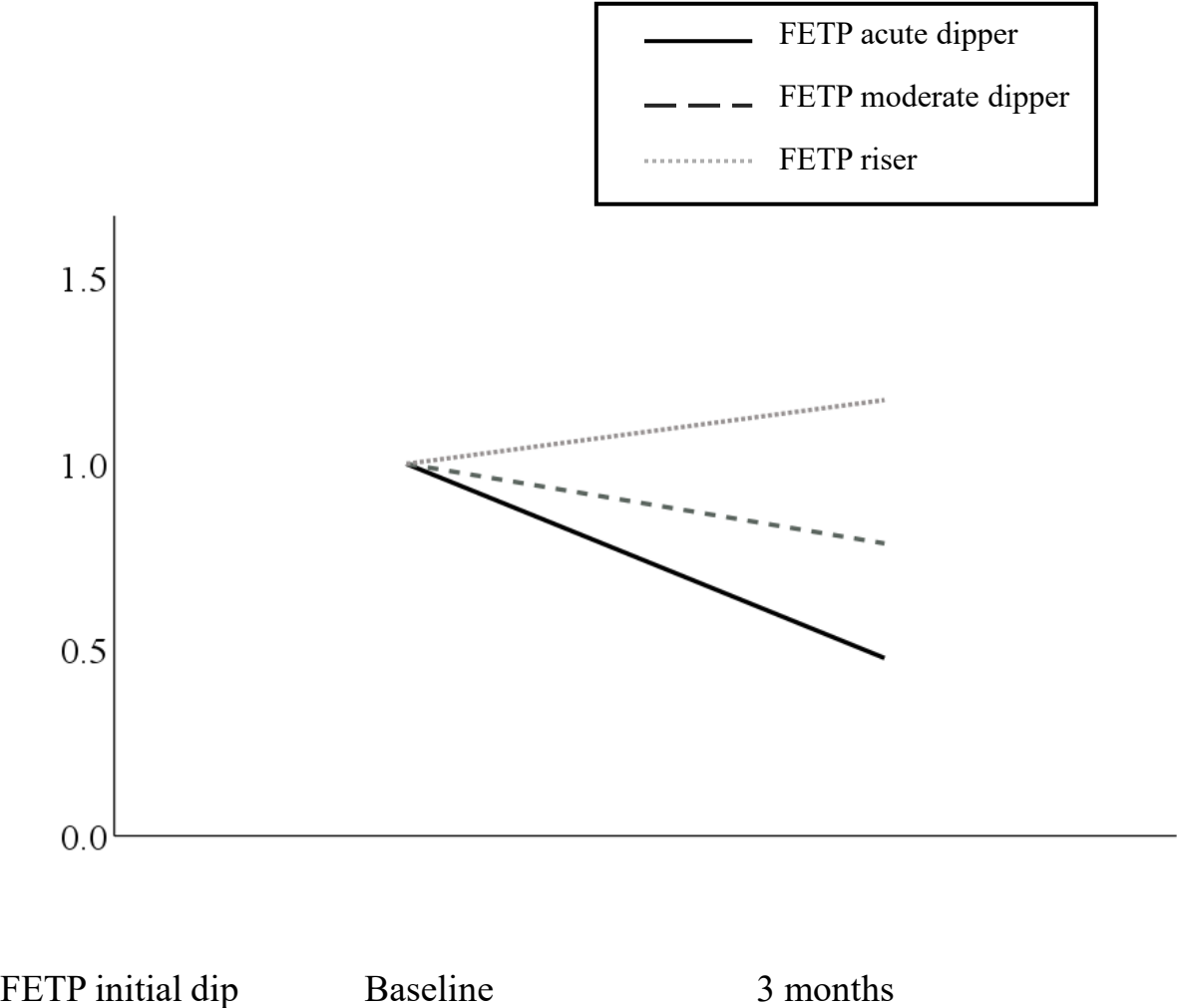

Supplementary Figure 1b

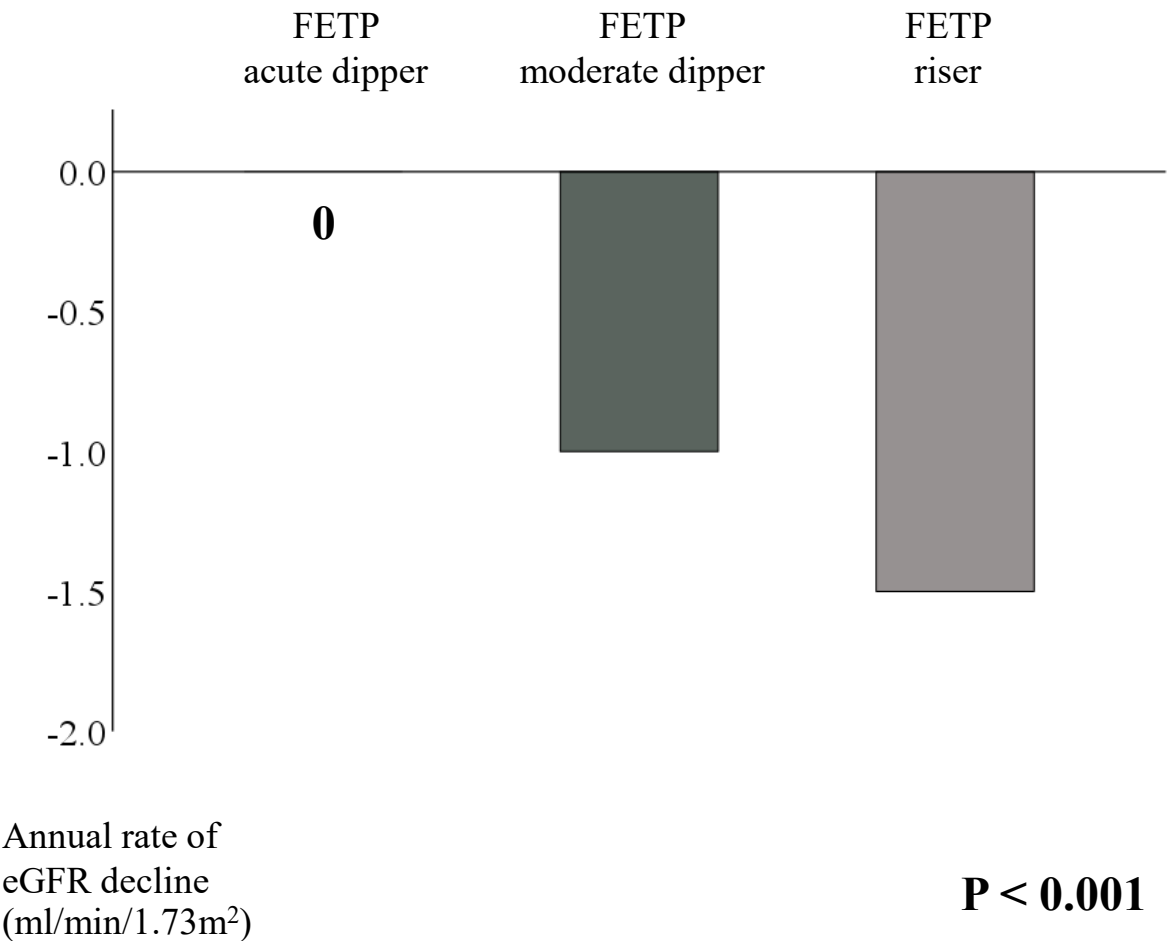

Supplement: sfaf209_Supplemental_Files [file sfaf209_supplemental_files.zip › Supplementary Figure1.pdf]

Supplementary Figure 2a

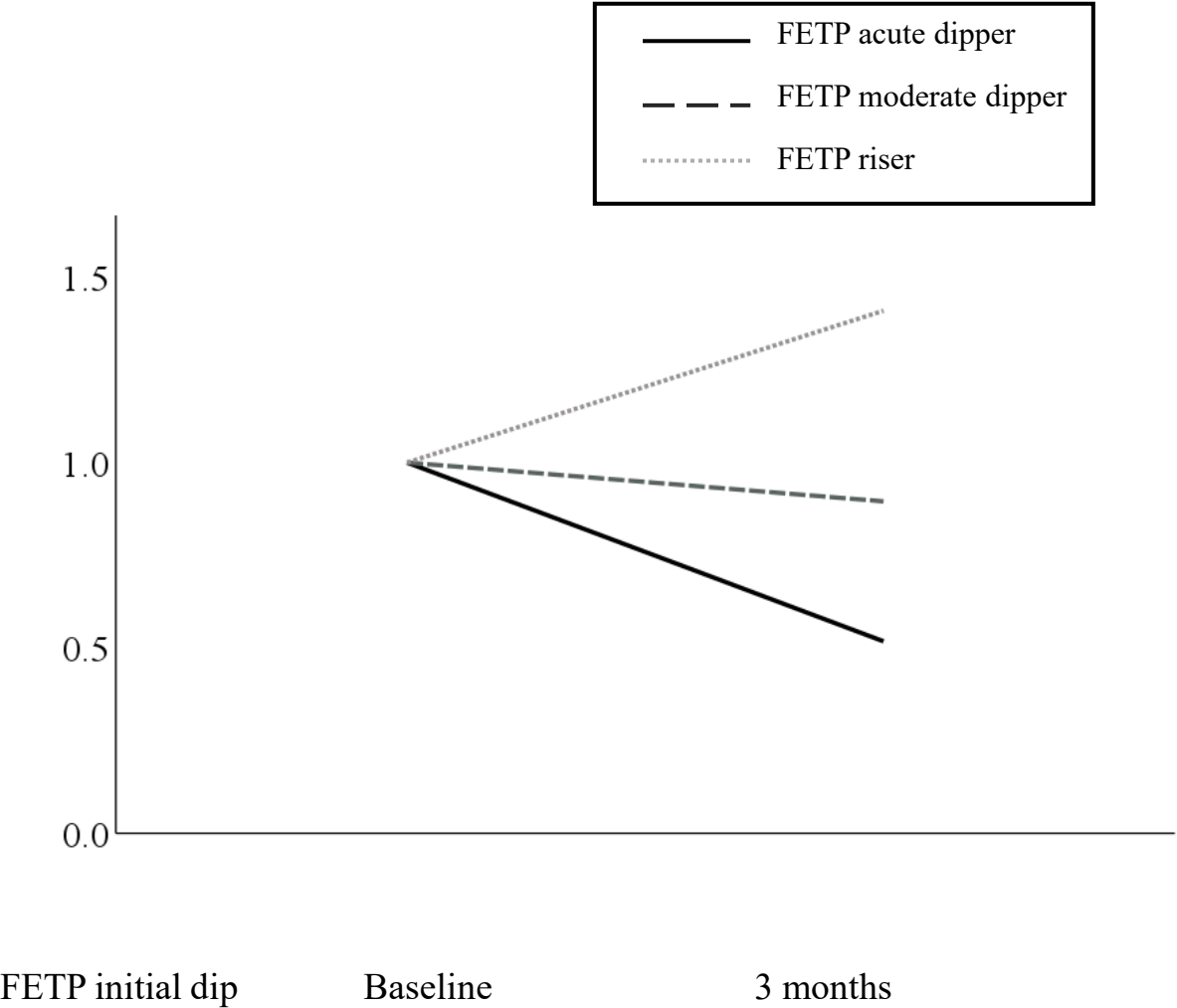

Supplementary Figure 2b

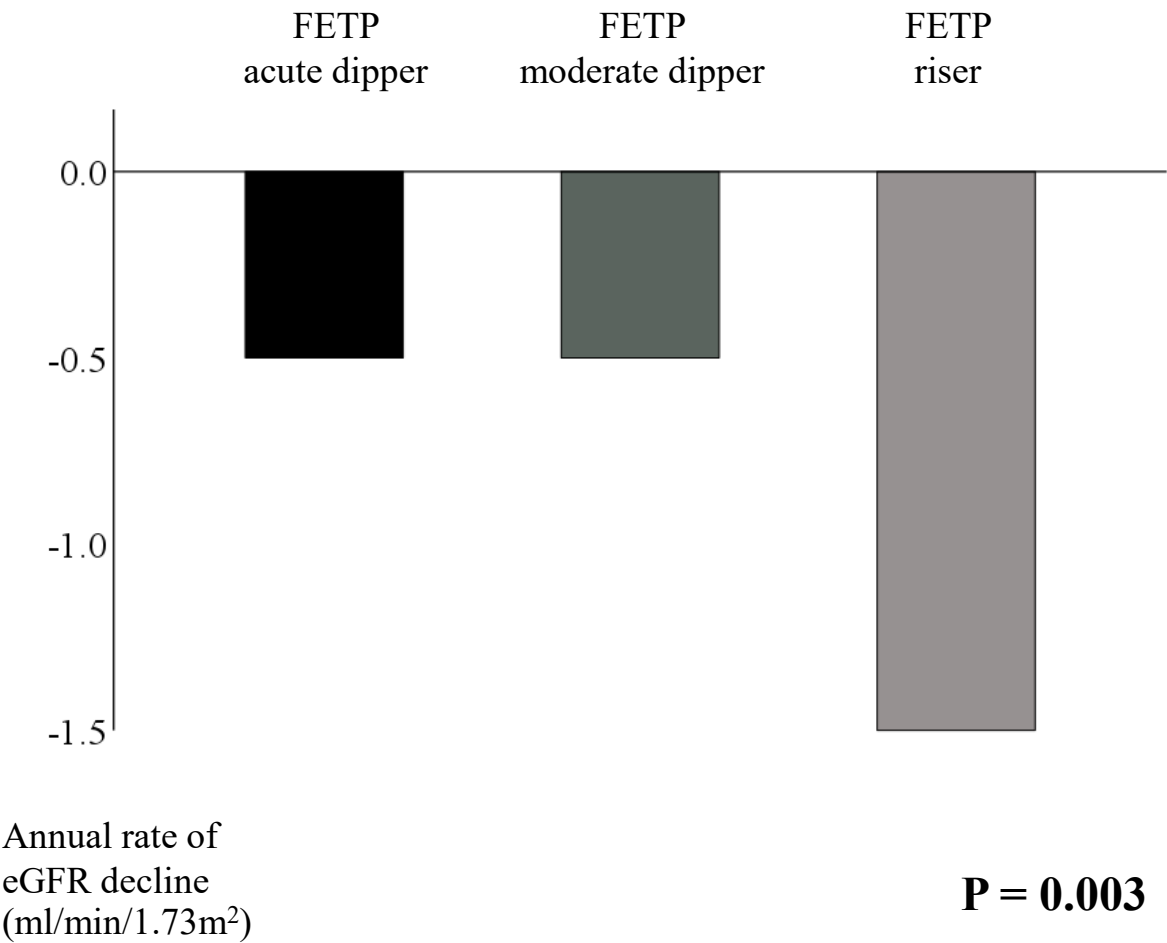

Supplement: sfaf209_Supplemental_Files [file sfaf209_supplemental_files.zip › Supplementary Figure2.pdf]
